# Acknowledgement to Reviewers of *Biomolecules* in 2014

**DOI:** 10.3390/biom5010001

**Published:** 2015-01-07

**Authors:** Biomolecules Editorial Office

**Affiliations:** MDPI AG, Klybeckstrasse 64, CH-4057 Basel, Switzerland

The editors of *Biomolecules* would like to express their sincere gratitude to the following reviewers for assessing manuscripts in 2014:

Admon, Arie
Alberti, Simon
Bach, Ingolf
Bai, Fan
Bardag-Gorce, Fawzia
Barducci, Alessandro
Bardwell, James
Bar-Nun, Shoshana
Bartels, Tim
Bastolla, Ugo
Berhow, Mark
Beyer, Katrin
Bianchi, Giada
Bjørås, Magnar
Blaber, Michael
Black, Jennifer D.
Blinov, Nikolay
Borella, Paola
Bour, Petr
Bracher, Andreas
Breitenbach, Michael
Bugliani, Marco
Caddeo, Carla
Caputo, Gregory A.
Carlson, Erin E.
Castano, Jose G.
Chaturvedula, Venkata S. P.
Chen, Chin Ho
Chiarelli, Roberto
Chou, Kuo-Chen
Citores, Lucía
Costa-Filho, Antonio
Criscitiello, Michael
Dahlmann, Burkhardt
Dallinger, Reinhard
Daniels-Wells, Tracy R.
Dargemont, Catherine
Das, Sabyasachi
Dionne, Marc S.
Dodevski, Igor
Eako, Hiroshi
Enenkel, Cordula
Farinha, Carlos
FINK, Katja
Förster, Friedrich
Freund, Christian
Fugueiredo-Pereira, Maria
Gaczynska, Maria
García-Fruitós, Elena
Gardner, Richard G.
Ghirlanda, Giovanna
Goto, Kaoru
Greenwood, Michael T.
Gupta, Vineet
Hagiwara, Ken'ichi
Harms, Mike
Hartl, Dominik
Hartmann-Petersen, Rasmus
Hassan, Mohamed
Henryk, Kozlowski
Hochrainer, Karin
Holz, Richard
Horovitz, Amnon
Hsu, Ellen
Huang, Haochu
Jackson, Edwin
Jazirehi, Ali
Johansson, Åsa
Jongco, Artemio
Kallenbach, Neville
Kampinga, Harm H.
Kato, Koichi
Kawahara, Masahiro
Kholodov, Yaroslav
Kipper, Matt
Kojima, Seiji
Koria, Piyush
Kosugi, Akihiko
Kovalenko, Ilya
Kurtz, Donald M. Jr.
Kusmierczyk, Andrew R.
Kwawabata, Takeshi
Liberles, David A.
Litvinov, Rustem
Lu, Xin
Lynen, Frederic
Malandrinos, M.
Mangani, Stefano
Maret, Wolfgang
Masucci, Maria
Matos, Paulo
Matouschek, Andreas
Millhauser, Glenn
Molteni, Carla
Murata, Shigeo
Nanda, Vik
Natalello, Antonino
Nilsson, Lars
Okochi, Mina
Osna, Natalia
Otzen, Daniel
Panasenko, Olesya O.
Parker, William
Pashov, Anastas
Peluffo, Daniel
Permyakov, Eugene
Platts, James
Ravid, Tommer
Riess, Olaf
Rizzarelli, Enrico
Roder, Heinrich
Rodríguez, Jose A.
Roelofs, Jeroen
Romero, Alejandro
Rovira, Carme
Rowinska-Zyrek, Magdalena
Rudick, Jonathan
Saeki, Yasushi
Sagui, Celeste
Sarhan, Dhifaf
Sarikas, Antonio
Schrum, Laura W.
Scudiero, Rosaria
Shafirovich, Vladimir
Sharova, Natalia
Shi, Zhengshuang
Shibasaki, Seiji
Shimizu, Shigeomi
Shoji, Ikuo
Sijts, Alice
Simal-Gándara, Jesús
Smith, David
Son, Deok-soo
Stemp, Eric D. A.
Stokke, Bjørn T.
Sukocheva, Olga
Sutton, Lesley-Ann
Tamaru, Yutaka
Tanabe, Kazuhito
Tashiro, Fumio
Tavan, Paul
Tiana, Guido
Toellner, Kai-Michael
Ulber, Roland
Vago, Riccardo
Vancurova, Ivana
Voit, Eberhard
Wallin, Stefan
Wang, Chu
Wang, Xuejun
Watanabe, Kunihiko
Watson, Corey
Wilson, Melanie
Witt, Stephan N.
Xu, Yan
Yahiroda, Hideki
Yan, Hongbin
Yin, Zhaojun
Yoshitaka, Matsumura
Yura, Kei
Zanotti, Giuseppe
Zeczycki, Tonya
Zhou, Yifa
Zou, Chunbin
Zou, Wen-Quan

